# Leaflet thrombosis in transcatheter aortic valve intervention: mechanisms, prevention, and treatment options

**DOI:** 10.3389/fcvm.2023.1249604

**Published:** 2023-10-06

**Authors:** Ofir Koren, Vivek Patel, Tarun Chakravarty, Hasan Jilaihawi, Aakriti Gupta, Shirin Sadri, Raj R. Makkar

**Affiliations:** ^1^ Cedars–Sinai Medical Center, Smidt Heart Institute, Los Angeles, CA, United States; ^2^ Bruce Rappaport Faculty of Medicine, Technion Israel Institute of Technology, Haifa, Israel; ^3^ Department of Medicine, Stanford University, Stanford, CA, United States

**Keywords:** TAVR, leaflet thrombosis, mechanism, prevention, prevalence, review

## Abstract

**Introduction:**

Transcatheter aortic valve intervention (TAVR) has emerged as a promising alternative to surgical aortic valve replacement for patients with severe aortic stenosis. However, leaflet thrombosis has raised concerns about the long-term durability and outcomes of TAVR. This study aims to provide an overview of the mechanisms, prevention strategies, and treatment options for leaflet thrombosis in TAVR.

**Clinical evidence:**

Leaflet thrombosis refers to the formation of blood clots on bioprosthetic valve leaflets, leading to impaired leaflet mobility, early valve degeneration and dysfunction, and potential clinical implications. While the mechanisms underlying thrombus formation on valve leaflets are not fully understood, several factors, such as altered blood flow patterns within valve neosinuses, prothrombotic surfaces, and patient-related causes, have been implicated. Two distinct entities have been identified, namely, hypoattenuated leaflet thickening and restricted leaflet motion. Their occurrence appears dynamic over time and is related to the valve type. Imaging, including transesophageal echocardiography and multidetector computed tomography, plays a crucial role in the diagnosis and follow-up of leaflet thrombosis.

**Prevention and treatment options:**

Preventing leaflet thrombosis requires a comprehensive and tailored approach involving identifying high-risk patients, close monitoring, and antithrombotic therapy. Antithrombotic therapy with dual antiplatelet agents or anticoagulation is commonly employed in TAVR patients, although the optimal regimen is yet to be defined. Novel antithrombotic agents, such as direct oral anticoagulants, are being investigated for their efficacy and safety in preventing leaflet thrombosis. When leaflet thrombosis is detected, treatment options include intensified antithrombotic therapy, valve-in-valve intervention, or balloon valvuloplasty. The long-term outcomes and impact of leaflet thrombosis on valve durability and patient prognosis are areas of ongoing research.

**Summary:**

Leaflet thrombosis in TAVR is a considerable complication affecting valve function and patient outcomes. Understanding the mechanisms underlying thrombus formation and implementing appropriate prevention strategies are essential for mitigating this risk. Treatment options aim to restore leaflet mobility and optimize valve performance. Further research is needed to establish standardized protocols for antithrombotic therapy, identify high-risk patient populations, and determine the long-term consequences of leaflet thrombosis on TAVR outcomes.

## Introduction

Leaflet thrombosis refers to the formation of both calcified and non-calcified thrombi on transcatheter heart valve (THV) leaflets, most commonly observed in transcatheter aortic valves (TAVs) made of bioprosthetic material (1). In 2015, Makkar et al. first recognized subclinical leaflet thrombosis (SLT) in transcatheter aortic valve intervention (TAVR). In their landmark study, they utilized multidetector computed tomography (CT) to assess leaflet abnormalities after balloon-expanding TAVR and demonstrated the occurrence of reduced leaflet motion associated with hypoattenuation opacities on CT in a subset of asymptomatic patients (2). This pioneering work prompted further investigation into its mechanisms, implications, and preventive and management strategies.

## Mechanism

**Figure 1:**
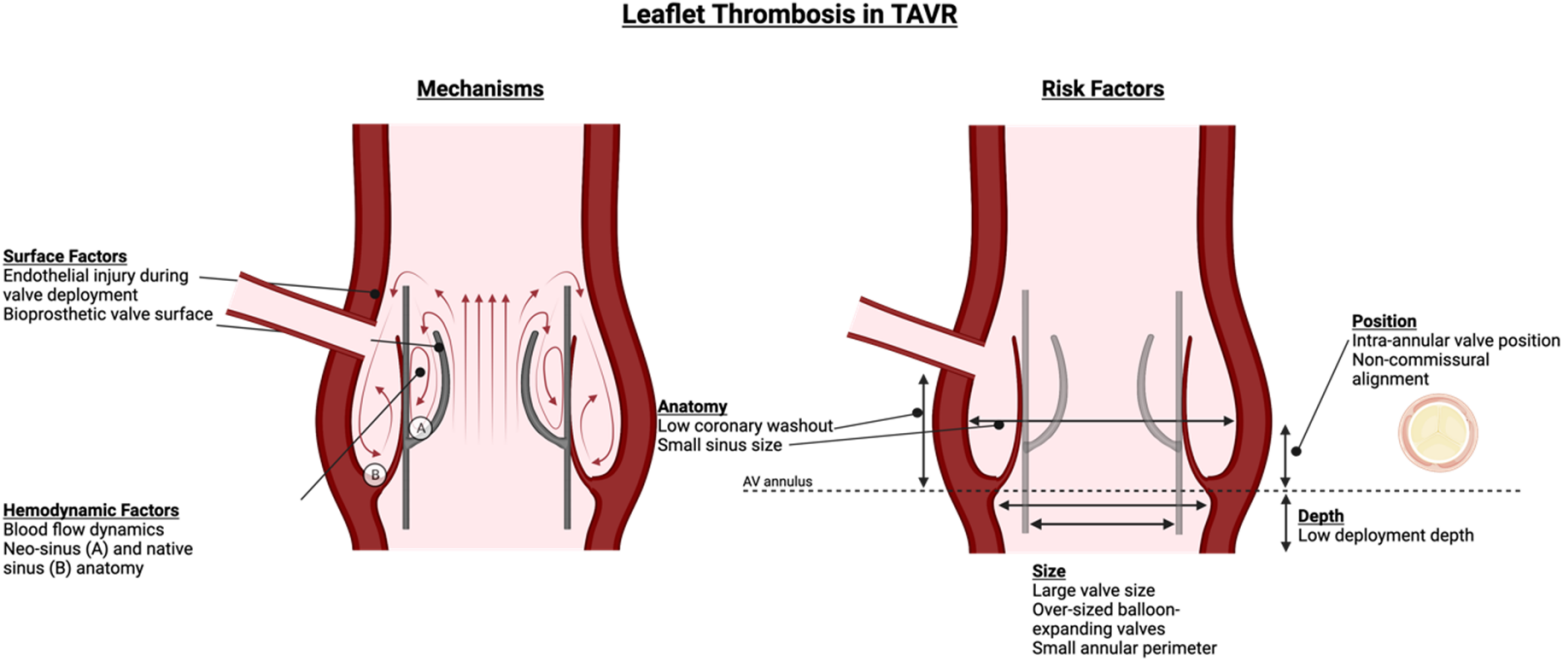
Leaflet thrombosis in TAVR—risk factors and mechanisms. Mechanisms of thrombus formation involve hemodynamic factors related to sinus anatomy and blood flow, as well as surface factors related to endothelial injury from valve deployment and valve surface. Risk factors are numerous and related to valve and annulus size and position as well as patient anatomy.

Pathophysiological mechanisms underlying leaflet thrombosis are still being elucidated. A modified Virchow's triad has been proposed, encompassing (i) hypercoagulability at the bioprosthetic surface, (ii) leaflet surface damage and/or endothelial injury during device deployment, and (iii) stasis and turbulent flow (3, 4). The formation of neosinuses between THV leaflets and the valve cage and between native leaflets and aortic wall creates semi-permeable barriers that lead to flow stasis and reduced washout forces (Figure 1) (5). Factors including larger-sized or overexpanded balloon-expandable valves, low-deployment depth of self-expanding valves (particularly CoreValve Evolut, Medtronic), intra-annular valve position, small annular perimeter, valve-in-valve (ViV) TAVR for stented surgical valves, non-commissural alignment, low coronary washout, small sinus size, and reduced flow can increase thrombosis risk (6–8). Valve maldeployment, which may occur due to factors such as extrinsic calcification or valve-in-valve deployment, can lead to incomplete cusp coaptation and excursion and increase thrombosis risk (9).

**Figure 2:**
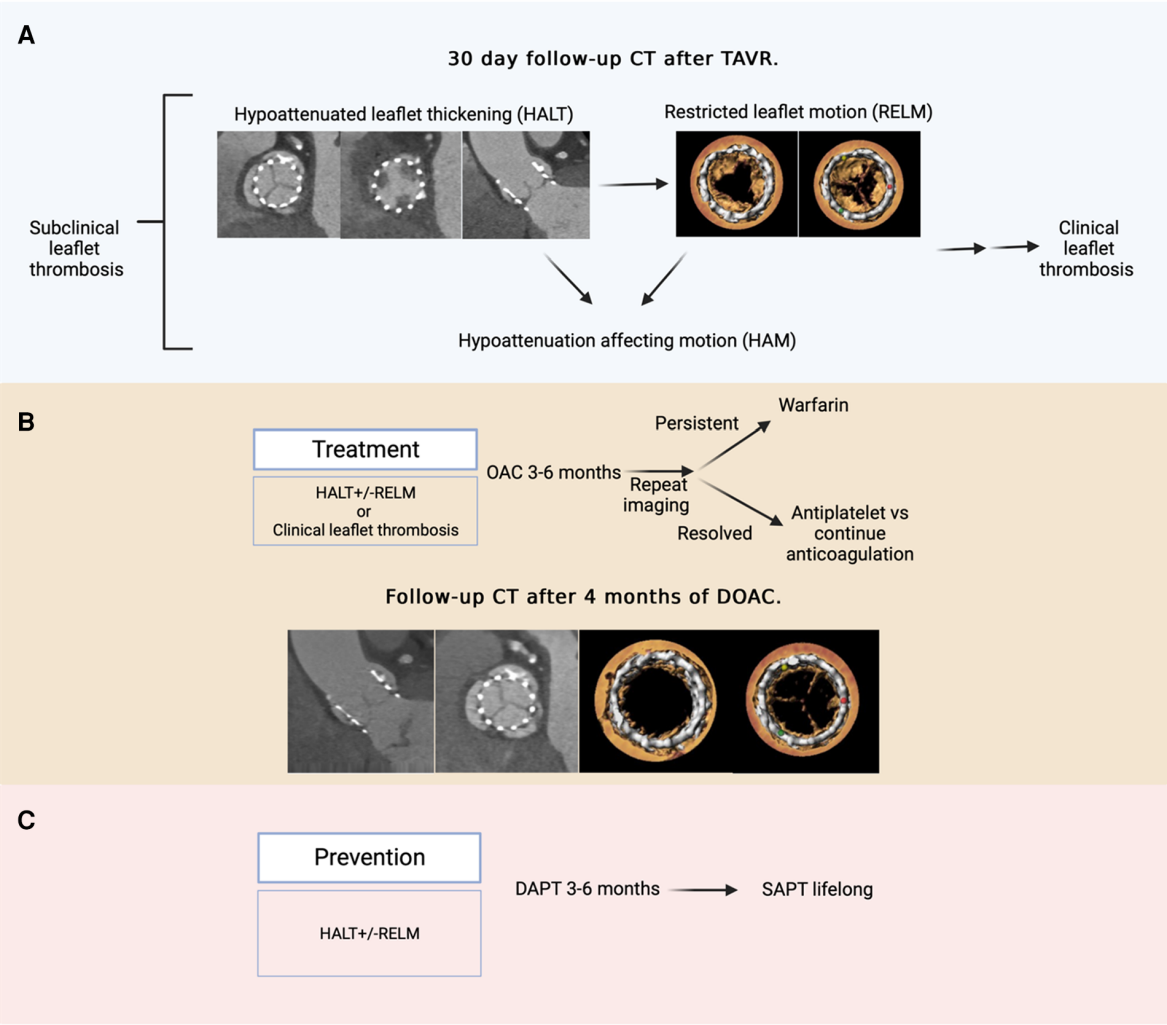
Hypoattenuated leaflet thickening, restricted leaflet motion, and management strategies. (**A**) An example of a 30-day follow-up CT following TAVR indicating HALT and RELM on all three leaflets and HAM of the non-coronary cusps. If left untreated, SLT may progress to clinical thrombosis, although the absolute risk of this is not yet fully understood. (**B**) Treatment of SLT involves 3–6 months of oral anticoagulation followed by repeat imaging to determine resolution and subsequent treatment strategy. A follow-up CT of the same patient following 4 months of direct oral anticoagulation (rivaroxaban 10 mg daily) treatment indicated a significant resolution of leaflet thrombosis with HALT observed only on the left coronary cusp and no RELM or HAM. (**C**) Prevention of SLT involves dual antiplatelet therapy for 3–6 months followed by lifelong single antiplatelet therapy.

Two distinct entities have been identified, namely, hypoattenuated leaflet thickening (HALT) and restricted leaflet motion (RELM). HALT refers to the abnormal thrombotic thickening of valve leaflets. RELM occurs secondary to HALT and impairs valve functionality. The co-occurrence of HALT and RELM defines hypoattenuation affecting motion (HAM) (Figure 2) (10, 11).

## Prevalence

Clinical leaflet thrombosis after TAVR is rare, affecting approximately 0.5% of TAVR patients, and can manifest as increased valve gradient and double the risk of cerebrovascular events and embolization to other arterial beds (12–14).

SLT occurs more commonly in up to 15% of TAVR patients and can vary dynamically over time based on the valve type and position. Inherent differences in THV compared with surgical valves, including mounting the valve within a rigid vs. more flexible stent, valve crimping with delivery, and native calcification, may alter thrombotic risk (15). In the PARTNER-3 sub-study involving 435 low-risk patients, HALT was more than twice as common after TAVR as compared with surgical aortic valve replacement (SAVR) at 30 days (13% vs. 5%, respectively), but the difference was no longer seen after 1 year. In the Evolut Low-Risk Leaflet Thickening or Immobility (LTI) sub-study evaluating the frequency of HALT in 503 patients undergoing TAVR using the self-expanding Evolut THV or SAVR, there was no difference in HALT between TAVR and SAVR at 30 days and 1 year (16,17). Notably, a recent study examining ViV TAVR demonstrated no difference in HALT or RELM between ViV and native-valve TAVR, despite historical concerns about the impact of ViV on sinus thrombosis (18).

## Diagnosis and clinical implications

Four-dimensional CT (4DCT) has emerged as a standard to identify HALT and RELM and is comparable to transesophageal echocardiology. Seen as hypoattenuating leaflet lesions on CT, HALT is assessed in diastole and requires visualization of leaflet coaptation. RELM is assessed in systole with maximal leaflet opening. Each leaflet is classified as mildly (<50%), moderately (50%–69%), or severely (70%–99%) restricted or immobile. HALT and >50% restriction signifies HAM (8).

The clinical significance of SLT is not well understood. Although the evidence is limited and conflicting, HALT may be associated with increased transient ischemic attack or stroke risk (RR: 2.6), with several studies showing no increase in cerebral ischemic events (19–21). If persistent or progressive, HALT minimally increases the valve gradient at 1 year compared with patients with no HALT or with spontaneously resolved HALT (17.8 vs. 12.7 mmHg, *p* = 0.04). An increase in the valve gradient appears concordant with the degree of HALT (17). Most studies have failed to demonstrate an association between HALT or RELM and heart failure symptomatology (22).

## Prevention and management

Early prevention strategies centered on antiplatelets, based on expert consensus and extrapolation from coronary stenting and historical bioprosthesis studies. Single antiplatelet therapy (SAPT) is preferred over dual antiplatelet therapy (DAPT) for long-term prevention (23, 24).

Oral anticoagulation (OAC)–based prevention strategies have produced mixed results. The GALILEO trial, which randomized 1,644 TAVR patients to either rivaroxaban (plus aspirin for 3 months) or antiplatelet (aspirin plus clopidogrel for 3 months) treatment, demonstrated a substantial reduction in HALT and RELM with the use of rivaroxaban. However, the trial was terminated early due to higher deaths and bleeding in the rivaroxaban treatment arm (25). The ATLANTIS study, which randomly assigned 1,500 TAVR patients to apixaban or standard care [vitamin K antagonist (VKA) or antiplatelet], demonstrated that apixaban vs. antiplatelet resulted in lower obstructive valve thrombosis but higher non-cardiovascular mortality with apixaban, while apixaban vs. VKA produced no difference in the primary outcome or safety (26). A small study randomizing TAVR patients to VKA plus aspirin or aspirin alone showed a reduction in the primary endpoint (7.0% vs. 26.5%), with VKA having no excess bleeding (27). Using routine OACs post-TAVR remains under debate, and future studies including comparisons of type and strength should be pursued.

The treatment selection for subclinical or clinical thrombosis requires careful assessment of thrombus burden and individual patient factors (Figure 2). Once confirmed on imaging, the preferred treatment involves OAC (apixaban, rivaroxaban, or warfarin) for 3–6 months until resolution. A reasonable approach is to monitor SLT in patients with a high bleeding risk because of its dynamic nature (17). Severe cases may require balloon valvuloplasty or transcatheter valve-in-valve implantation (17, 28).

## Summary

Leaflet thrombosis is a dynamic phenomenon that relies on local and systemic factors to develop. The best prevention strategies are still under debate, arising from concerns about OAC bleeding risk with or without antiplatelet therapy. Further investigation is needed to improve the prevention strategies and better understand the clinical implications and progression to valve degeneration.
